# Unresponsive shock due to amlodipine overdose: An unexpected cause

**DOI:** 10.15171/jcvtr.2018.43

**Published:** 2018-12-05

**Authors:** Shailesh Kumar, Devyani Thakur, Ritesh Kumar Gupta, Alka Sharma

**Affiliations:** Department of Medicine, Dr RML Hospital and PGIMER, Delhi, India

**Keywords:** Vasopressor, Shock, Vasopressor, HIET

## Abstract

Amlodipine is a dihydropyridine calcium channel blocker which is widely used as an antihypertensive drug. Amlodipine overdose have been infrequently reported with occurrence of serious complications and even death in a few cases. We report an interesting case of a young lady who presented with refractory shock with acute kidney injury, which did not respond to therapy despite optimal fluid replacement and vasopressor support. The etiology of shock could not be ascertained and the patient was questioned again to elucidate the missing clue in the history. It was finally revealed that the patient had consumed 900 mg of amlodipine in a suicide bid, for her poor performance in academics. The targeted therapy in the form of IV calcium and hyperinsulinemia-euglycemia therapy (HIET) was started and the patient dramatically improved with shock reversal and improvement in renal function.

## Introduction

The ever-burgeoning number of patients with cardiovascular and hypertensive diseases have resulted in a concomitant increase in the prescription of calcium channel antagonists (CCA), which gives them a potential for abuse, accidental or intentional. CCAs directly inhibit the opening of voltage-gated L-type calcium channel and prevent calcium influx into myocardial and vascular smooth muscle cells. Calcium influx initiates excitation–contraction coupling, sino-atrial node depolarization in the myocardium and maintains vascular and gastrointestinal (GI) smooth muscle tone.^1^ CCAs also inhibit L-type calcium channels in pancreatic islet cells, reducing insulin secretion and resulting in hyperglycaemia and reduced cardiac glucose utilization.^1^ CCA toxicity may produce hypotension, bradycardia, metabolic acidosis and shock, which in many cases, is refractory to inotropes and vasopressors.^2^ Amlodipine is a dihydropyridine(DHP) CCA which has a convenient once daily dosing due to its low metabolic clearance. There are several cases of amlodipine overdosage reported in the literature, many with lethal outcomes.^3-5^ Here we report a case of refractory shock which later turned out to be due to amlodipine intoxication and its subsequent successful management with IV calcium and HIET therapy.

## Case Report

A 22 year old lady, without any co-morbidities, presented with history of multiple episodes of vomiting three days back, reduced urine output for 3 days and shortness of breath since 2 days. On examination, the patient was conscious, oriented and afebrile. She had a blood pressure of 70/50 mm Hg and a pulse rate of 108/min. She was tachypnoeic with an oxygen saturation of 85% on room air. Her neck veins were engorged and she had bilateral pedal edema. Systemic examination revealed bilateral pleural effusion and ascites. On preliminary blood investigations there was evidence of acute kidney injury (AKI) with a serum creatinine of 1.8. Arterial blood gas analysis was suggestive of Type I respiratory failure. Her ECG showed sinus tachycardia and the chest roentgenogram confirmed bilateral pleural effusion. The patient was resuscitated with intravenous (IV) fluid bolus, oxygen inhalation and dual inotropes (noradrenaline and dopamine). The shock remained unresponsive even after 24 hrs of admission therefore inotropes were stepped up and empirical antibiotics were added. The diagnostic pleural tap revealed a transudative picture. Blood culture and urine culture were sterile and serum pro-calcitonin level was normal. At this stage the family was questioned again and it was revealed that the patient had attempted suicide due to poor academic performance by consuming 900 mg of amlodipine (90 tablets of 10 mg each). The targeted management was started with IV calcium gluconate infusion at 1 g/h, 50% dextrose IV bolus followed by regular insulin 1 U/kg bolus and then regular insulin at 0.5 U/kg/h with IV dextrose 25 g/h. The patient showed dramatic symptomatic improvement. Inotropes were tapered and stopped within 4 hours. Renal function improved with restoration of adequate urine output within 24 hours. The patient was discharged 3 days later with stable vitals and was advised to follow-up in psychiatry OPD.

## Discussion

Amlodipine is a commonly prescribed anti-hypertensive agent, which belongs to DHP group of calcium channel blockers. CCAs overdose presents usually with hypotension and bradycardia, but DHP intoxication (like amlodipine) causes arterial vasodilation and reflex tachycardia.^1,2^ With an increase in the ingested dose of DHP ,this selectivity is lost and myocardium and conducting system gets affected resulting in reduced cardiac output and bradycardia. This combination of vasodilation and decreased cardiac output causes hypotension. The patients may also present with pulmonary edema due to myocardial depression. They might have an altered mental status and are often hyperglycemic due to reduced insulin secretion.^1,2^

Management of CCA toxicity focuses on restoration of cardiac function and systemic blood pressure. In a hypotensive patient, optimal fluid resuscitation is needed with addition of inotropes, if required. Gastric lavage is recommended especially if the patient presents within a couple of hours of ingestion. Intravenous calcium is of utility for CCA overdose and can be used either as a bolus or a continuous infusion.^6^ Glucagon with its positive inotropic and chronotropic effects has been shown to be useful in multiple animal studies but not in human trials.^7^ Sodium bicarbonate is another potentially useful therapy in the treatment of CCA overdose. In an acidotic environment, CCA binding to the L-type calcium channel is enhanced. Therefore correction of the acidosis may improve the hemodynamic status.^2,6,7^

In recent years hyperinsulinemia-euglycemia therapy (HIET) has gained wide acceptance as a part of the treatment strategy for CCA toxicity.^8-10^ CCA over-dosage results in hyperglycemia from reduction in the insulin production due to blockage of L-type calcium channels in the pancreas. When hypoinsulinemia and acquired insulin resistance occur, the myocardium is unable to utilise glucose thereby reducing its contractility and causing hypotension. HIET reverses the cardiovascular collapse by improving myocardial utilization of carbohydrates and clearance of lactic acid and other glycolytic byproducts. In addition, insulin has direct positive inotropic activity that may contribute to its clinical effects.^8^ Hypoglycemia and hypokalemia are the main adverse effects of HIE therapy; therefore, serum glucose and electrolytes should be closely monitored.

This case is of immense clinical significance because CCA overdose, whether intentional or accidental, can be lethal and therefore one needs to be aware of its pathophysiology and clinical presentation. Moreover even in this modern world of technology, the conventional art of eliciting a good history can help clinicians to arrive at a final diagnosis, as was demonstrated in our case. And lastly, even in the absence of a suggestive history, CCA overdose should be considered as a differential in patients with unexplained refractory shock.

## Ethical approval

An informed consent was taken from the patient for publishing this case report.

## Competing interests

All authors declare no competing financial interests exist.

## Funding

The authors did not receive any specific funding for this work.

## References

[1] Graudins A, Lee HM, Druda D (2015). Calcium channel antagonist and beta-blocker overdose: antidotes and adjunct therapies. Br J Clin Pharm.

[2] DeWitt CR, Waksman JC (2004). Pharmacology, Pathophysiology and Management of Calcium Channel Blocker and Beta Blocker Toxicity. Toxicol Rev.

[3] Johansen SS, Genner J (2003 Sep). A fatal case of amlodipine poisoning. Journal of Clinical Forensic Medicine.

[4] Sklerov JH, Levine B, Ingwersen KM, Aronica-Pollack PA, Fowler D (2006). Two cases of fatal amlodipine overdose. J Anal Toxicol.

[5] Ghosh S, Sircar M (2008). Calcium channel blocker overdose: Experience with amlodipine. Indian J Crit Care Med.

[6] Salhanick SD, Shannon MW (2003). Management of Calcium Channel Antagonist Overdose. Drug Saf.

[7] Kerns W (2007). Management of β-adrenergic blocker and calcium channel antagonist toxicity. Emerg Med Clin North Am.

[8] Boyer EW, Shannon M (2001). Treatment of calcium-channel–blocker intoxication with insulin infusion. N Engl J Med.

[9] Yuan TH, Kerns WP, Tomaszewski CA, Ford MD, Kline JA, Kline J (1999). Insulin-Glucose as Adjunctive Therapy for Severe Calcium Channel Antagonist Poisoning. J Toxicol.

[10] Azendour H, Belyamani L, Atmani M, Balkhi H, Haimeur C (2010). Severe amlodipine intoxication treated by hyperinsulinemia euglycemia therapy. J Emerg Med.

